# Supplementary material for: Knockdown of Antisense Noncoding Mitochondrial RNA Reduces Tumorigenicity of Patient-Derived Clear Cell Renal Carcinoma Cells in an Orthotopic Xenograft Mouse Model
Source: Cancers (Basel). 2024 Feb 19;16(4):830. doi: 10.3390/cancers16040830 (PMC10886546; doi:10.3390/cancers16040830)
Supplement: Supplementary file 1 [file cancers-16-00830-s001.zip › cancers-2828696-Supplementary Figures/Supplementary Figure S1.pdf]

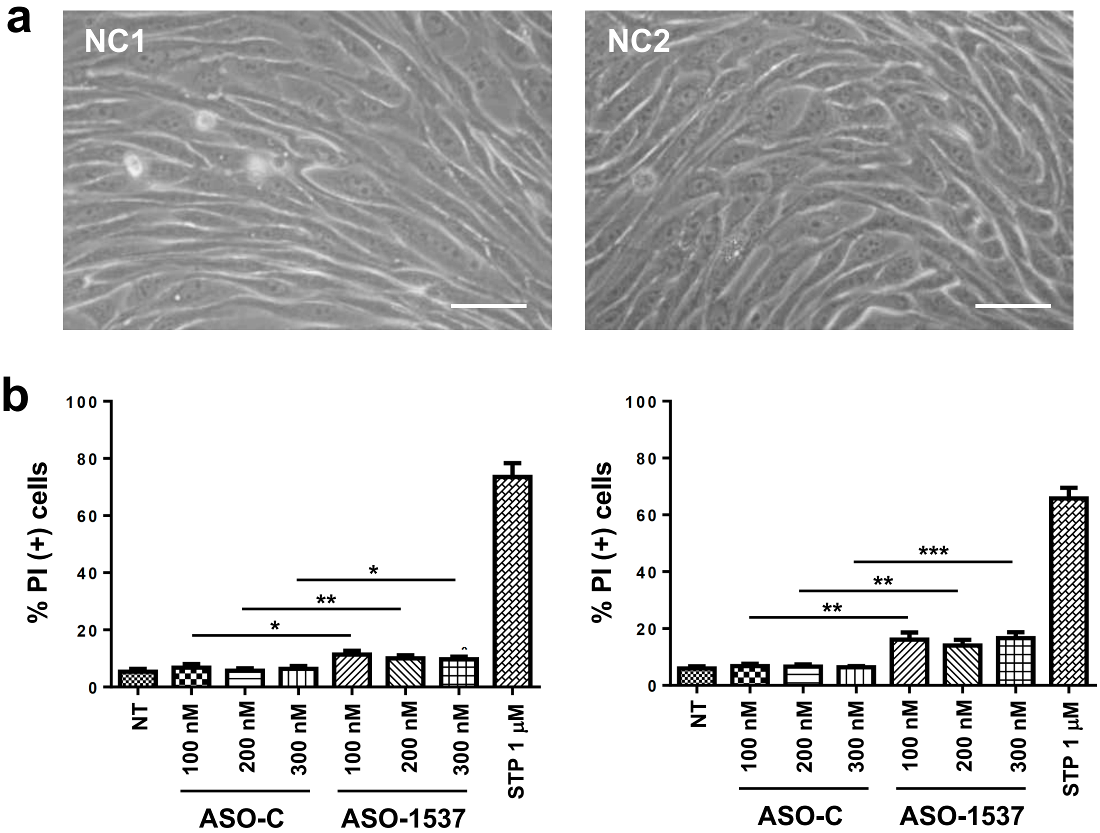

Figure S1. Normal kidney epithelial cultures are resistant to ASO-1537 treatment. Two individual cultures, NC1 and NC2, from non-tumor tissue obtained from ccRCC patients (**a**) were transfected with ASO-C or ASO-1537 at 100, 200 and 300 nM for 48 h, stained with PI and analyzed by flow cytometry. Although slightly higher than ASO-C, ASO-1537 elicited only a modest % of cell death even at the highest concentration. Staurosporin (STP) was used as a positive control for cell death. *$p<0.05$; **$p<0.01$; ***$p<0.001$. Bars in **a** = 100 µm.
