# Supplementary material for: Knockdown of Antisense Noncoding Mitochondrial RNA Reduces Tumorigenicity of Patient-Derived Clear Cell Renal Carcinoma Cells in an Orthotopic Xenograft Mouse Model
Source: Cancers (Basel). 2024 Feb 19;16(4):830. doi: 10.3390/cancers16040830 (PMC10886546; doi:10.3390/cancers16040830)
Supplement: Supplementary file 1 [file cancers-16-00830-s001.zip › cancers-2828696-Supplementary Figures/Supplementary Figure S2.pdf]

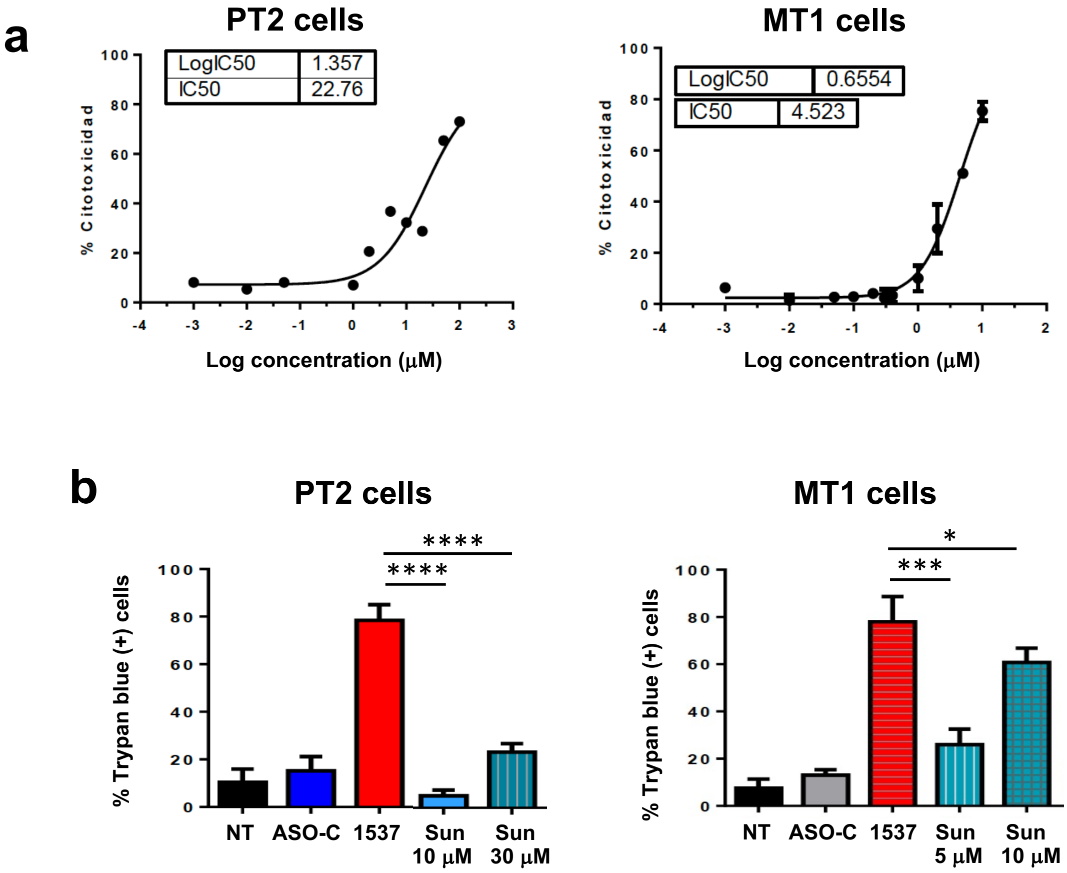

Figure S2. Comparison of ASO-1537 and Sunitinib on PT2 and MT1 cells. (a) Sulforhodhamine assay for determination of citotoxicity of Sunitinib on PT2 and MT1 primary ccRCC cultures at 72 h. **(b)** Trypan blue exclusion assay for cell death of PT2 and MT1 cells comparing NT cells, ASO-C, ASO-1537 and different concentrations of Sunitinib at 48 h. *p<0.05; ***p<0.001; ****p<0.0001.
