# Supplementary material for: Knockdown of Antisense Noncoding Mitochondrial RNA Reduces Tumorigenicity of Patient-Derived Clear Cell Renal Carcinoma Cells in an Orthotopic Xenograft Mouse Model
Source: Cancers (Basel). 2024 Feb 19;16(4):830. doi: 10.3390/cancers16040830 (PMC10886546; doi:10.3390/cancers16040830)
Supplement: Supplementary file 1 [file cancers-16-00830-s001.zip › cancers-2828696-Supplementary Figures/Supplementary Figure S3.pdf]

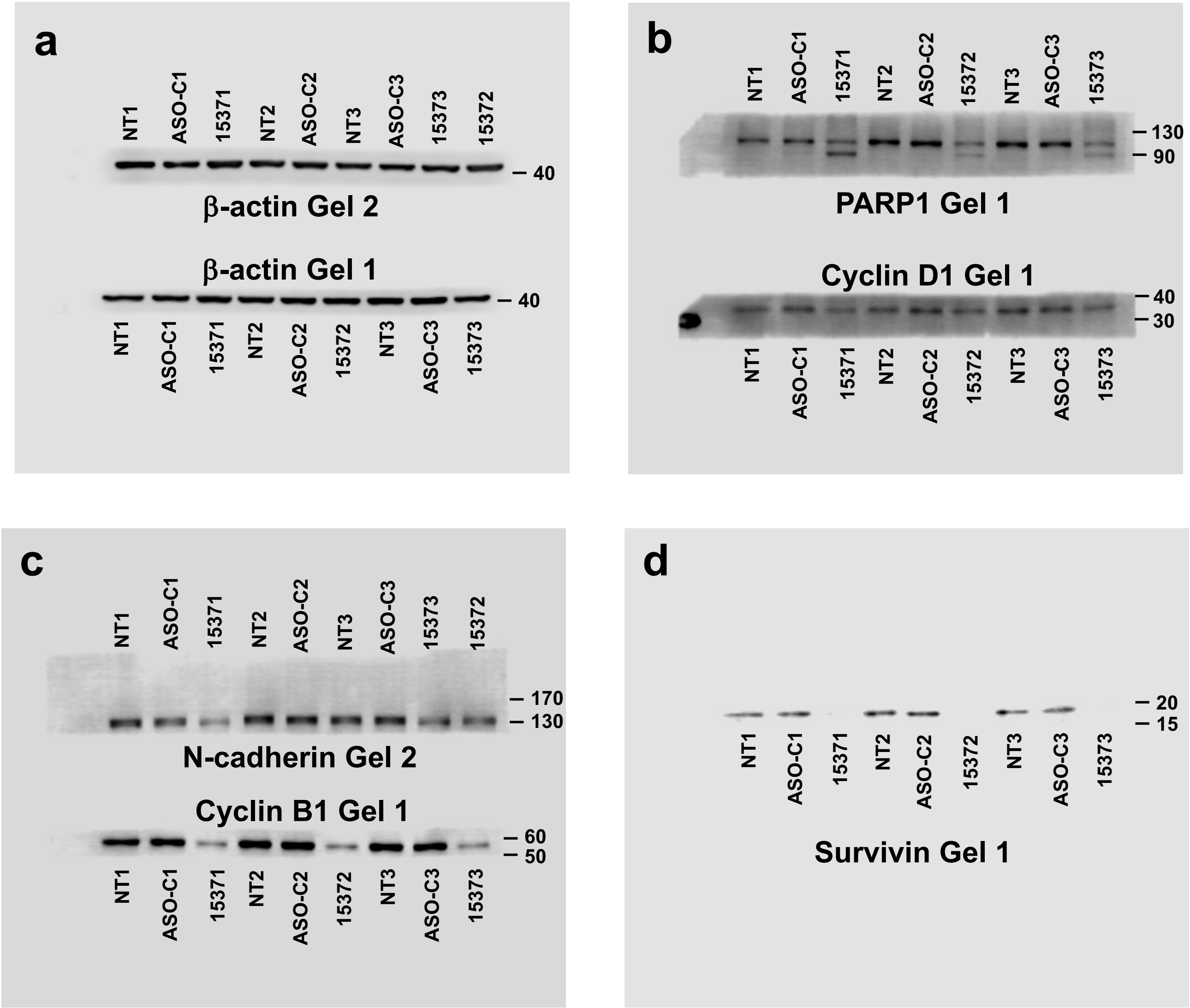

Figure S3. Original Western blot scanner images for Figures 2d and 3b. Images correspond to 2 different blots, termed "Gel 1 and Gel 2". Membranes from each gel were cropped before antibody incubation**. (a)** β-actin blot for Gel 2 (above; N-cadherin, Fig. 3b) and Gel 1 (below; Cyclins B1 and D1 (Fig. 3b), Survivin and PARP1 (Fig. 2d). **(b)** Gel 1 above, PARP1 (Fig. 2d); below, cyclin D1 (Fig. 3b). **(c)** Above, Gel 2 (N-cadherin, Fig. 3b); below, Gel 1, cyclin B1 (Fig. 3b). **(d)** Gel 1, survivin (Fig. 2d). Migration position of adjacent protein MW standards are shown on the right. Note: there was an error in the loading order of Gel 2 (a and c; see labels above the blots). For presentation purposes, the order was digitally switched in the main figure (3b).
