# Supplementary material for: Knockdown of Antisense Noncoding Mitochondrial RNA Reduces Tumorigenicity of Patient-Derived Clear Cell Renal Carcinoma Cells in an Orthotopic Xenograft Mouse Model
Source: Cancers (Basel). 2024 Feb 19;16(4):830. doi: 10.3390/cancers16040830 (PMC10886546; doi:10.3390/cancers16040830)
Supplement: Supplementary file 1 [file cancers-16-00830-s001.zip › cancers-2828696-Supplementary Figures/Supplementary Figure S4 .pdf]

a

	Survivin	β-actin	Surv/β-act	Surv/β–act normalized to NT
NT1	5,285,134	3,685,284	1.43411851	1
ASO-C 1	6,463,719	3,596,284	1.797332747	1.253266542
1537-1	50,364	3,883,698	0.012968053	0.009042525
NT2	6,072,548	4,184,527	1.451191019	1
ASO-C 2	6,074,891	4,320,820	1.405957897	0.968830346
1537-2	0	4,354,820	0	0
NT3	5,552,719	4,546,062	1.221434947	1
ASO-C 3	5,539,376	4,709,477	1.17621893	0.962981232
1537-3	6,121	3,866,941	0.001582905	0.001295939

b

	Uncleaved PARP1	Cleaved PARP1	% Cleaved PARP1/Total PARP1
NT1	11,732,907	57,192	0.485084985
ASO-C 1	14,606,522	603,477	3.967633397
1537-1	7,643,510	10,192,551	57.14575096
NT1	15,429,978	61,192	0.395012126
ASO-C 2	17,183,593	113,607	0.656794163
1537-2	5,346,560	2,240,125	29.52705958
NT1	14,749,865	20,950	0.141833744
ASO-C 3	13,515,401	150,607	1.102055553
1537-3	3,535,267	3,502,995	49.77073886

Figure S4. Densitometry readings and plotted values of Western blots of Fig. 2d. (a) Survivin, **(b)** Cleaved and uncleaved PARP1. Readings were obtained using ImageJ and values were normalized to non-treated samples (NT).
