# Supplementary material for: Knockdown of Antisense Noncoding Mitochondrial RNA Reduces Tumorigenicity of Patient-Derived Clear Cell Renal Carcinoma Cells in an Orthotopic Xenograft Mouse Model
Source: Cancers (Basel). 2024 Feb 19;16(4):830. doi: 10.3390/cancers16040830 (PMC10886546; doi:10.3390/cancers16040830)
Supplement: Supplementary file 1 [file cancers-16-00830-s001.zip › cancers-2828696-Supplementary Figures/Supplementary Figure S5.pdf]

a

	Cyc B1	β-actin	Cyc1/β-act	CycB1/β-act normalized to NT
NT1	8,523,610	3,685,284	2.312877379	1
ASO-C 1	9,789,075	3,596,284	2.721997206	1.176887815
1537-1	2,578,154	3,883,698	0.663839979	0.287019098
NT2	11,019,660	4,184,527	2.633430254	1
ASO-C 2	10,608,347	4,320,820	2.455169852	0.932308668
1537-2	3,514,397	4,354,820	0.807013149	0.30644941
NT3	10,458,933	4,546,062	2.3006578	1
ASO-C 3	9,054,518	4,709,477	1.92261646	0.835681195
1537-3	2,347,912	3,866,941	0.607175543	0.263913887

b

	Cyc D1	β-actin	CycD1/β-act	CycD1/β-act normalized to NT
NT1	10,260,167	3,685,284	2.78409127	1
ASO-C 1	10,319,418	3,596,284	2.86946693	1.03066554
1537-1	4,492,276	3,883,698	1.15670065	0.415467935
NT2	7,574,175	4,184,527	1.81004329	1
ASO-C 2	7,978,054	4,320,820	1.84642128	1.020097859
1537-2	3,923,134	4,354,820	0.90087168	0.497707256
NT3	10,109,116	4,546,062	2.22370834	1
ASO-C 3	9,336,288	4,709,477	1.98244688	0.891504899
1537-3	4,238,225	3,866,941	1.09601491	0.492877097

c

	N-cadh	β-actin	N-cadh/βact	N-cadh/β-act normalized to NT
NT1	12,810,468	3,685,284	3.476114188	1
ASO-C 1	7,617,276	3,596,284	2.118096346	0.609328759
1537-1	214,435	3,883,698	0.055214128	0.015883865
NT2	11,556,468	4,184,527	2.761714287	1
ASO-C 2	12,008,761	4,320,820	2.779278239	1.006359801
1537-2	2,848,326	4,354,820	0.654062854	0.236832194
NT3	9,483,397	4,546,062	2.086068558	1
ASO-C 3	11,113,933	4,709,477	2.359908117	1.131270642
1537-3	2,674,912	3,866,941	0.691738509	0.331599125

Figure S5. Densitometry readings and plotted values of Western blots of Fig. 3b. (a) Cyclin B1, **(b)** Cyclin D1, **(c)** N-cadherin. Readings were obtained using ImageJ and values were normalized to non-treated samples (NT).
