# Supplementary material for: Knockdown of Antisense Noncoding Mitochondrial RNA Reduces Tumorigenicity of Patient-Derived Clear Cell Renal Carcinoma Cells in an Orthotopic Xenograft Mouse Model
Source: Cancers (Basel). 2024 Feb 19;16(4):830. doi: 10.3390/cancers16040830 (PMC10886546; doi:10.3390/cancers16040830)
Supplement: Supplementary file 1 [file cancers-16-00830-s001.zip › cancers-2828696-Supplementary Figures/Supplementary Figure S7.pdf]

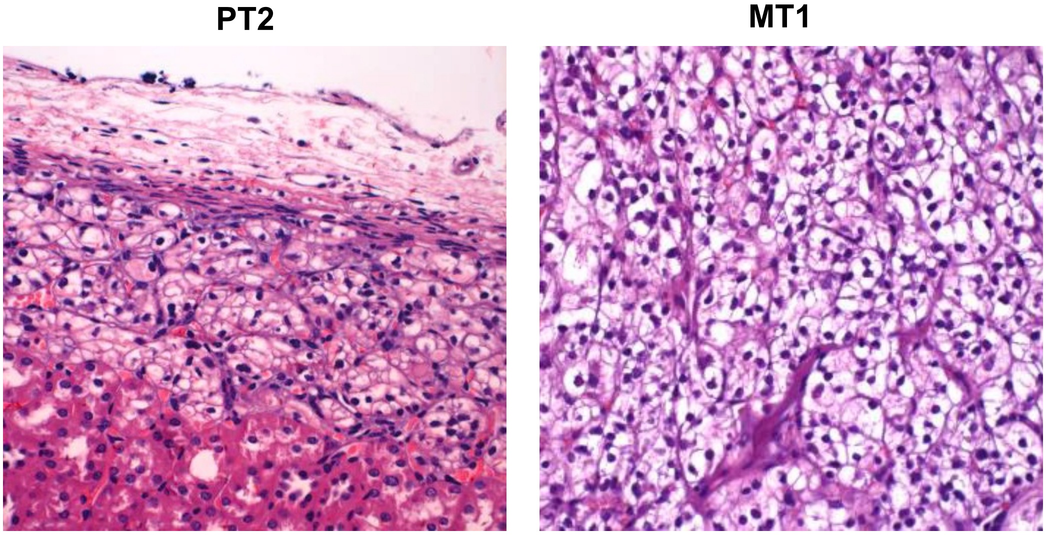

Figure S7. H&E-stained sections of subcapsular patient-derived ccRCC tumors in NOD-SCID mice, at 6 weeks post-inoculation. Left, PT2; Right, MT1. Both tumors display the clear cytoplasm characteristic of ccRCC. 40X magnification.
