# Supplementary material for: Knockdown of Antisense Noncoding Mitochondrial RNA Reduces Tumorigenicity of Patient-Derived Clear Cell Renal Carcinoma Cells in an Orthotopic Xenograft Mouse Model
Source: Cancers (Basel). 2024 Feb 19;16(4):830. doi: 10.3390/cancers16040830 (PMC10886546; doi:10.3390/cancers16040830)
Supplement: Supplementary file 1 [file cancers-16-00830-s001.zip › cancers-2828696-Supplementary Figures/Supplementary Figure S8 .pdf]

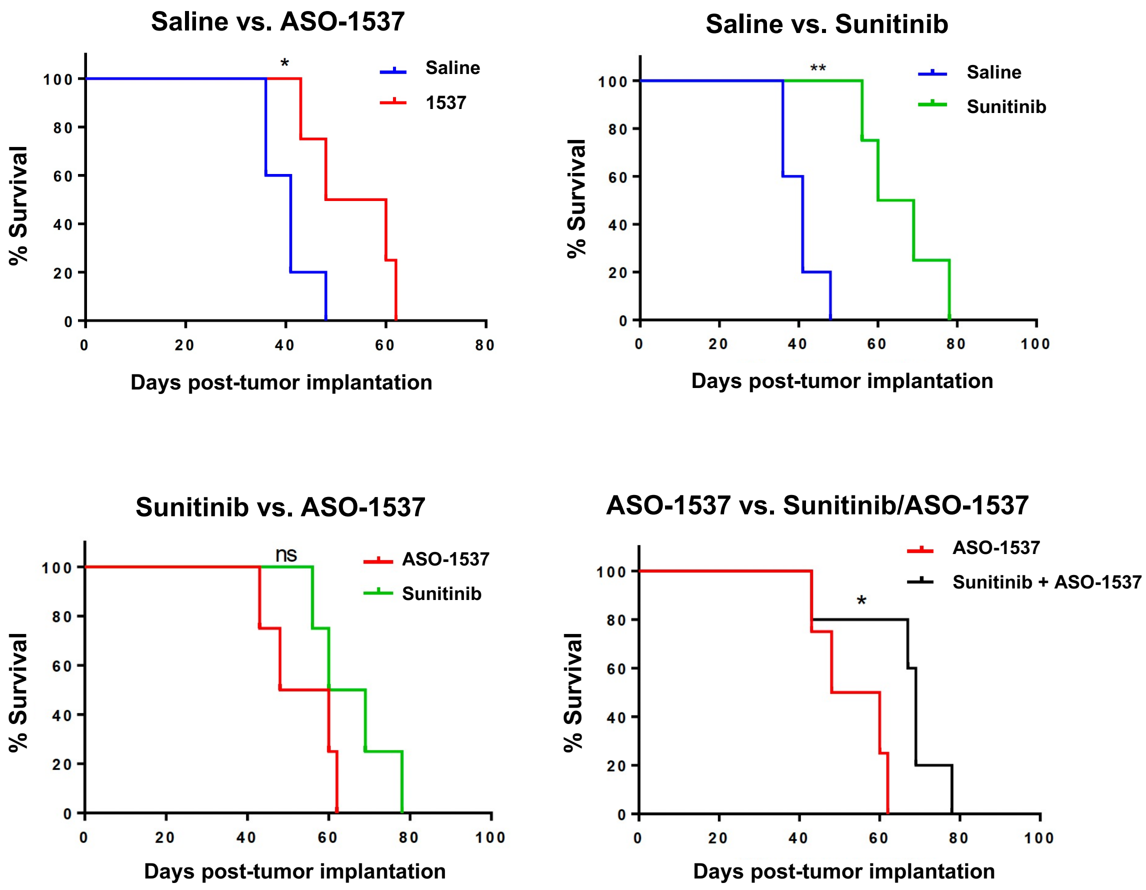

Figure S8. Survival curves of NOD/SCID mice implanted subcutaneously with MT1 tumor fragments. For better clarity, data is separated to compare 2 conditions in each graph. Kaplan-Meier plots; *$p < 0.5$; **$p < 0.1$; ns, non-significant.
